# Predictive value of coronary calcifications for future cardiac events in asymptomatic patients with diabetes mellitus: A prospective study in 716 patients over 8 years

**DOI:** 10.1186/1471-2261-8-27

**Published:** 2008-10-10

**Authors:** Alexander Becker, Alexander W Leber, Christoph Becker, Franz von Ziegler, Janine Tittus, Ines Schroeder, Gerhard Steinbeck, Andreas Knez

**Affiliations:** 1 Department of Cardiology, Ludwig-Maximilians-University, Munich, Germany; 2 Department of Clinical Radiology, Ludwig-Maximilians-University, Munich, Germany

## Abstract

**Background:**

To establish an efficient prophylaxis of coronary artery disease reliable risk stratification is crucial, especially in the high risk population of patients suffering from diabetes mellitus. This prospective study determined the predictive value of coronary calcifications for future cardiovascular events in asymptomatic patients with diabetes mellitus.

**Methods:**

We included 716 patients suffering from diabetes mellitus (430 men, 286 women, age 55.2 ± 15.2 years) in this study. On study entry all patients were asymptomatic and had no history of coronary artery disease. In addition, all patients showed no signs of coronary artery disease in ECG, stress ECG or echocardiography. Coronary calcifications were determined with the Imatron C 150 XP electron beam computed tomograph. For quantification of coronary calcifications we calculated the Agatston score. After a mean observation period of 8.1 ± 1.1 years patients were contacted and the event rate of cardiac death (CD) and myocardial infarction (MI) was determined.

**Results:**

During the observation period 40 patients suffered from MI, 36 patients died from acute CD. The initial Agatston score in patients that suffered from MI or died from CD (475 ± 208) was significantly higher compared to those without cardiac events (236 ± 199, p < 0.01). An Agatston score above 400 was associated with a significantly higher annualised event rate for cardiovascular events (5.6% versus 0.7%, p < 0.01). No cardiac events were observed in patients with exclusion of coronary calcifications. Compared to the Framingham risk score and the UKPDS score the Agatston score showed a significantly higher diagnostic accuracy in the prediction of MI with an area under the ROC curve of 0.77 versus 0.68, and 0.71, respectively, p < 0.01.

**Conclusion:**

By determination of coronary calcifications patients at risk for future MI and CD could be identified within an asymptomatic high risk group of patients suffering from diabetes mellitus. On the other hand future events could be excluded in patients without coronary calcifications.

## Background

Diabetes mellitus is one of the most common diseases in the industrialized countries. The prevalence is expected to rise over 5% in the next 10 years [1]. Thereby patients with diabetes mellitus show an increased risk of cardiovascular events [2]. Independent of concomitant cardiovascular risk factors diabetic patients possess an up to fourfold risk of cardiovascular events, equivalent to the risk of nondiabetic patients with a history of coronary artery disease [3]. In addition, the fatality rate of CAD is significantly higher in diabetic patients [4]. Overall 65% of diabetic patients die because of cardiovascular diseases.

To assess the risk of future events in this high risk population, several score systems based on concomitant risk factors and diseases have been introduced. Thereby the diagnostic accuracy in the prediction of future events is still limited [5-7]. Beside the scores based on epidemiological studies electron beam computed tomography offers the possibility to detect and quantify the amount of coronary calcium (CAC). In histopathologic studies coronary calcifications have shown to be a sensitive marker of early stages of coronary atherosclerosis [8,9]. Furthermore, the amount of coronary calcifications correlates to the extent of coronary atherosclerosis and coronary stenosis [10,11]. Several studies have already shown the association of future cardiovascular events with an elevated amount of coronary calcifications. In addition, the exclusion of coronary calcification is associated with a strong negative predictive value for future cardiovascular events. Still it is questionable, whether the determination of CAC adds an additional prognostic and clinical value in a high risk population of patients [12-14]. Therefore, we concentrated in this prospective study on asymptomatic patients suffering from diabetes mellitus and assessed the prognostic value of CAC over an observation period of 8 years. In addition, the results were compared to conventional models of risk stratification, the Framingham cardiovascular risk score, and the UKPDS risk engine [5,15].

## Methods

### Study protocol

The research protocol was approved by the local Clinical Institutional Review Board and complies with the declaration of Helsinki. From January 1998 to April 1999 we asked 938 asymptomatic patients with Diabetes mellitus type 2 referred to our clinic for a preventive medical check up to take part in this prospective study. 776 patients agreed to undergo EBCT scanning and follow up-interview and were included in this study after giving written consent. All patients had no history of CAD and were asymptomatic regarding CAD. In addition no signs of CAD could be found in ECG, stress ECG and echocardiography. Thereby Stress ECG was not available in 18 patients, echocardiography in 12 patients.

### Risk factors

Diagnosis of diabetes mellitus was assured in all patients by determination of glucose in fasting state as described by the definition of the World Health Organization [16]. Additional cardiovascular risk factors were assessed by personal interview and screening of medical records. In every patient arterial blood pressure (three times after 10 minutes rest), LDL cholesterol level, HDL cholesterol level, and triglyceride level were determined in the fasting state in our hospital. Arterial hypertension was defined as systolic blood pressure above 140 mmHg or diastolic blood pressure above 90 mmHg. Family history was measured by personal interview and defined as coronary heart disease in male first degree relative <55 years; coronary heart disease in female first degree relative <65 years.

Based on cardiovascular risk factors and concomitant diseases we calculated the risk for future cardiac death or nonfatal myocardial infarction based on the Framingham population and the UKPDS risk engine [17]. According to both scores patients were assigned to one of the following risk groups: low risk (event rate < 10%), intermediate risk (event rate > 10% and < 20%), and high risk (event rate > 20%).

### EBCT scanning procedure

EBCT scans were performed using the Imatron C-150 XP scanner (Imatron, South San Francisco, California, USA) in the high resolution mode. Images were acquired ECG-triggered at 80 percent of the R-R interval during one end-inspiratory breathholding period. Slice thickness was 3 mm, scan time 100 ms. A field of view of 26 cm was chosen. A total of 40 slices were obtained covering the whole heart. Coronary calcifications were defined as lesions with a density > 130 HU in more than 2 adjacent pixels. For quantification of coronary calcium we calculated the Agatston score, which is the product of the lesions area and a factor from 1 to 4 representing the peak density of the lesion [18]. EBCT scans were evaluated without knowledge of patient's characteristics. The patients were unaware of their Agatston score.

### Clinical follow-up

Study patients were contacted after an observation time of 8 years after the CT scan. An interims analysis was performed after 4 years. Cardiovascular events were assessed using a standardized telephone interview. In case of hospital admission or further cardiologic examinations the patient's medical records were reviewed for complaints of chest discomfort, dyspnea, myocardial infarction, and coronary revascularization.

### Clinical endpoints

The study endpoints were cardiac death (CD) and myocardial infarction (MI) as hard events and coronary revascularization (CR), coronary artery bypass graft (CABG) or percutaneous transluminal coronary angioplasty (PTCA), as soft events. MI was defined as the presence of at least 2 of the following: prolonged chest pain leading to hospital admission, specific ECG changes, elevation of serum creatine kinase levels up to twice the upper limit with an elevated creatine kinase-MB fraction or troponin level. Death due to coronary artery disease was considered when death was proved to be due to coronary atherosclerosis by autopsy, occurred within 1 hour after onset of prolonged severe chest pain, or occurred during hospital admission because of MI. Coronary interventions had to be confirmed by reports of the performing physician.

### Statistics

Statistical analyses were performed using the SPSS software package (version 10.0, SPSS Inc. Chicago, Illinois). Agatston score was expressed as mean score ± standard deviation except where indicated. Because of the non-normality, statistical analysis was performed on the base 10 log of the transformed Agatston score + 1. To compare score values in different risk groups, we used the Wilcoxon signed rank test for unpaired data. A p-value under 0.05 was considered to indicate statistical significance. We performed univariate Cox regression analysis to calculate hazard ratio and 95 percent confidence interval of cardiac death and MI in dependence of cardiovascular risk factors (patients without cardiovascular risk factors served as the reference group), age, sex, and Agatston score (patients without coronary calcifications served as the reference group). To account for the inflation of the type I error due to multiple testing, we performed the Bonferroni adjustment. The significance level was set at 0.05/4 = 0.0125 and 0.05/2 = 0.025.

In addition, Cox proportional hazards survival curves showing the estimated event free survival determined by the Cox regression model were calculated for patients in different score categories [19]. The results were adjusted for differences in age and sex. To verify the assumption of proportional hazards we performed an analysis of the Kaplan-Meier curves as described by Hosmer and Lemeshow.

To demonstrate the discriminatory power of Agatston score, UKPDS score, and Framingham score in prediction of cardiovascular events the area under the receiver-operating characteristic (ROC) curve was determined. ROC curve analysis was performed as follows: Sensitivity was plotted as a function of the false positive rate (1-specificity) for predicting cardiovascular events. An area under the curve of 1.0 represents a perfect test with 100 percent sensitivity and 100 percent specificity, whereas an area of 0.5 represents a random discrimination. Areas under the curve above 0.7 might indicate a reasonably good clinical test in combination with a sufficient diagnostic accuracy (sensitivity, specificity, negative and positive predictive value). Different ROC curves were compared using the method of Hanley and McNeil [20].

## Results

716 individuals (430 men and 280 women, age 55.2 ± 15.2 years) of the initially 776 included patients completed the follow up. 32 patients died of non-cardiac death, 28 patients could not be contacted for a follow-up examination. There was no difference in age, risk factor distribution, and Agatston score between these patients that completed the follow up and those that did not finish the study. The mean observation time was 8.1 ± 1.1 years (range 7.1 to 9.4 years). Patients' characteristics and therapies are given in table 1. The mean number of risk factors per person was 3.0 ± 1.3 in men and 2.8 ± 1.1 in women (range 1 – 5), see table 1.

**Table 1:** Baseline characteristics of 716 patients included in the study

	all	men	women
number (n)	716	430	286
age (years)	55.2 ± 15.2	55.5 ± 15.2	54.8 ± 15.2
no additional risk factor, n (%)	143 (20)	83 (20)	60 (21)
arterial hypertension, n (%)	372 (52)	249 (58)	123 (43)
treated, BP < 140/90 n (%)	125 (17)	77 (18)	48 (17)
treated, BP > 140/90 n (%)	140 (20)	96 (22)	44 (15)
untreated, BP > 140/90 n (%)	107 (15)	76 (18)	31 (11)
hyperlipidemia or statin therapy, n (%)	301 (42)	184 (44)	117 (41)
statin therapy, cholesterol < 5 mmol/l n (%)	99 (14)	61 (14)	38 (13)
statin therapy, cholesterol > 5 mmol/l n (%)	124 (17)	70 (16)	54 (19)
untreated, cholesterol > 5 mmol/l n (%)	78 (11)	53 (12)	25 (9)
MI in family history, n (%)	451 (63)	271 (63)	186 (65)
smoking, n (%)	243 (34)	151 (35)	94 (33)
HbA1c (%)	7.5	7.6	7.5
average number of risk factors	2.9	3.0	2.8
body mass index (kg/m^2^)	27.1 ± 6.1	28.3 ± 5.8	25.0 ± 4.9
duration of Diabetes (years)	6.5 ± 2.3	6.4 ± 2.2	6.6 ± 2.3
microalbuminuria n (%)	64 (9)	41 (9)	23 (8)
proteinuria n (%)	41 (6)	25 (6)	16 (6)
peripheral neuropathy n (%)	104 (15)	60 (14)	44 (15)
retinopathy n (%)	145 (20)	90 (21)	55 (19)
atrial fibrillation n (%)	95 (13)	61 (14)	24 (12)
insulin only n (%)	105 (15)	62 (15)	43 (15)
insulin + oral agent n (%)	109 (15)	70 (16)	39 (14)
oral agent only n (%)	502 (70)	298 (69)	204 (71)

### Distribution of Agatston score

The mean Agatston score was 288 ± 153 (range 0 – 2849, median 576, quartile rank 145 and 877), 319 ± 187 for men, and 241 ± 157 for women, p = 0.02. The score increased from 34 ± 11 (range 0 – 99) for patients under 40 years to 418 ± 178 (range 0 – 2849) for patients older than 70 years. In 107 patients coronary calcifications could be excluded (score 0), 302 patients showed an Agatston score above 400. In all age groups the mean score in men was significantly higher than in women. In patients with additional arterial hypertension (252 ± 160, range 0 – 701, p = 0.03), and hyperlipidemia (439 ± 256, range 0 – 699, p = 0.01) the average Agatston score was significantly higher than in patients without additional cardiovascular risk factors (124 ± 74, range 0 – 430).

### Cardiac events

36 patients died from CD (0.6% annualised rate), 40 patients suffered from MI (0.7% annualised rate) and 87 patients (64 patients PTCA and 23 patients CABG) underwent coronary revascularization (1.5% annualised rate). There was no significant difference between men and women in all event rates, p = 0.12

No patient with exclusion of cardiovascular calcifications suffered from MI or CD during the observation period, only two patients with exclusion of coronary calcifications underwent coronary revascularization. The event rates of patients with exclusion of coronary calcifications were significantly lower compared to patients with coronary calcifications, p = 0.009, see table 2. In patients with scores above 400 the annualised rates were significantly higher for all events (p = 0.011).

**Table 2:** Event rates for coronary revascularisation (CR), myocardial infarction (MI), and cardiac death (CD) in all patients, patients with Agatston score 0, and Agatston score above 400: Total number of events during observation period and annualised rate

	All patients	no event	CR		MI + CD		all events	
	n	n	n	% per year	n	% per year	n	% per year
Patients (n)	716	553	87	1.5	76	1.3	163	2.8
men (n)	430	332	53	1.5	45	1.3	98	2.8
Women (n)	286	224	31	1.3	31	1.3	62	2.7
Score 0	107	105	2	0.2	0	0.0	2	0.2
Score > 400	302	165	71	2.9 *	66	2.7 *	137	5.6 *
number of risk factors	2.9	2.7	2.8		2.9		2.9	
age (years)	55.7 ± 13.0	55.3 ± 13.2	57.4 ± 12.4		56.9 ± 13.1		57.1 ± 12.9	
Agatston score	288 ± 153	236 ± 199	447 ± 228 *		475 ± 208 *		459 ± 219 *	
Framingham risk score	9.6 ± 3.4	8.6 ± 3.4	13.1 ± 3.9 *		13.7 ± 4.1 *		13.3 ± 3.8 *	
UKPDS Score	9.6 ± 2.8	8.4 ± 3.6	13.7 ± 4.0 *		14.0 ± 4.3 *		13.8 ± 4.1 *	

Score parameters (UKPDS score, Framingham score, and Agatston score) in patients with and without cardiovascular events * indicates a significant higher event rate and a significant difference in score, respectively, † indicates a significant lower event rate (p < 0.05) compared to the event rate in all patients (first row).

**Figure 1:**
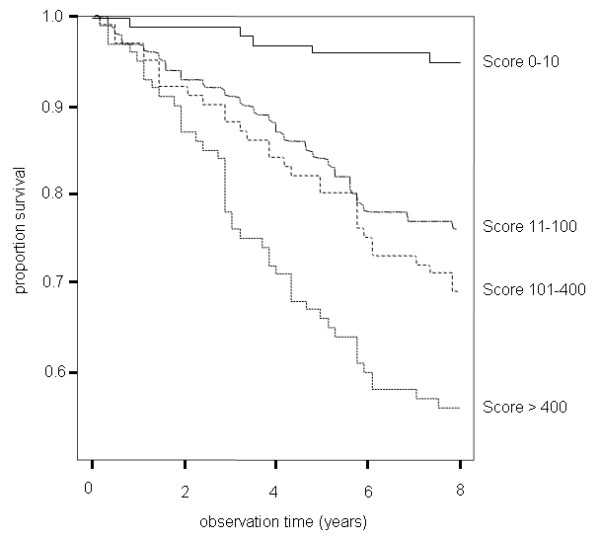
Cox proportional hazards survival curves showing the cumulative event free survival for patients with scores from 0–10, 11–100, 101–400, and above 400.

Figure 1 shows the association between calcium score categories and event free survival, the results were adjusted for differences in age and sex. The cumulative incidence increased continuously form 3.9% for patients with scores between 0 – 10 to a cumulative incidence of 42.7% for patients with scores above 400.

### Scores in patients with MI or CD

The mean Agatston score of patients that died from CD or suffered from MI was significantly higher than in patients without cardiovascular events, 475 ± 208 compared to 236 ± 199, p = 0.009. In addition, patients that underwent CR showed a significantly higher Agatston score of 447 ± 228 compared to patients without cardiac event, 236 ± 199, p = 0.007. Framingham risk score and UKPDS score were significantly higher in patients with cardiovascular events. There was no significant difference in age or average number of risk factors between patients with or without cardiac events (table 2).

**Figure 2:**
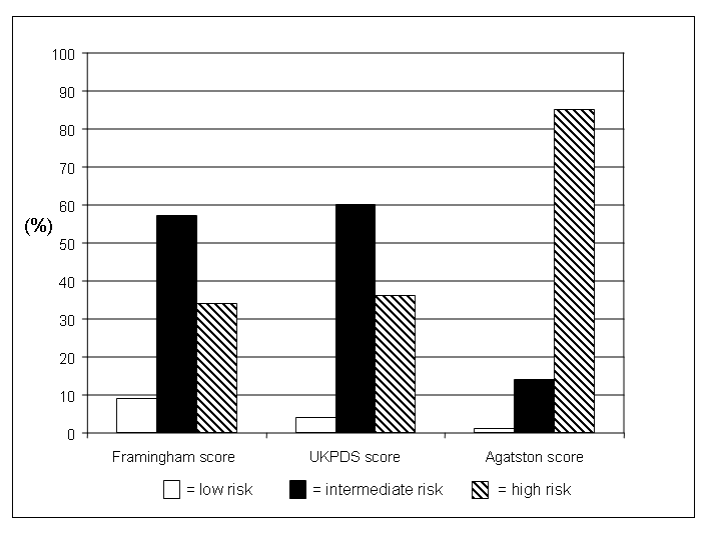
Risk stratification according to UKPDS score, Framingham score, and Agatston score in patients suffering from myocardial infarction (n = 76).

Figure 2 depicts the distribution of Framingham score, UKPDS score, and Agatston score in patients with MI. According to Framingham score (57%) and UKPDS score (60%) most patients with MI were considered as intermediate risk patients. 34% and 36%, respectively, were assigned to the high risk group. 9% (Framingham score) and 4% (UKPDS score) of patients with MI would have been considered as low risk patients. The proportion of patients with MI and an Agatston score above the 75^th ^percentile added up to 86%, 13% had an Agatston score below the 75^th ^and above the 25^th ^percentile, and 1% had an Agatston score below the 25^th ^percentile.

### Relative Risk of MI

The relative risk for MI and CD in dependence of additional cardiovascular risk factors was lowest for patients with smoking, 2.14, and increased with 2.47 for patients with arterial hypertension up to 2.75 for patients with hyperlipidemia. The relative risk for MI and CD was increased in patients with diabetes associated diseases, 1.76 in patients with retinopathy, 1.36 in patients with microalbuminuria, and 1.76 in patients with proteinuria. An increase of Framingham score and UKPDS score was associated with an increase of relative risk, 6.0 for a ten unit increase of Framingham score and 7.1 for a ten unit increase of UKPDS score. The highest relative risk of 14.2 for MI or CD was calculated for patients with Agatston scores above 400 (table 3). In addition table 4 shows the relative risk of cardiovascular events for different categories of calcifications: We found a continuously increasing risk of cardiovascular events with increasing calcium scores, the relative risk increased from 3.1 (2.8 women) for patients with scores from 0 – 10 to 32.8 (50.0 women) for patients with scores above 400.

**Table 3:** Univariable Cox proportional hazards model predicting the combined primary endpoint myocardial infarction and cardiac death (MI and CD) in dependence of cardiovascular risk factors and Agatston scores above the 75. percentile

	relative risk	95% CI	p value
no risk factors	1	-	-
arterial hypertension	2.47 *	2.21–2.81	0.005
smoking	2.14 *	2.01–2.33	0.005
hyperlipidemia	2.75 *	2.49–3.19	0.006
microalbuminuria	1.36	1.30–1.47	0.151
proteinuria	1.71 *	1.56–1.97	0.018
familiy history	1.45 *	1.28–1.73	0.012
duration of diabetes	1.51	1.39–1.69	0.013
retinopathy	1.76 *	1.60–2.04	0.013
HbA1C	1.89 *	1.69–2.19	0.009
Framingham risk score	6.0 *	4.9–7.8	0.004
UKPDS Score	7.1 *	6.0–8.9	0.003
Agatston score > 400	14.2 *	12.7–16.8	0.001

* indicates a significant difference (p < 0.05) compared to the patient group without cardiovascular risk factors and without coronary calcifications, respectively.

**Table 4:** Cox proportional hazards model predicting the combined primary endpoint (MI and CD) in different score groups for men a) and women b) adjusted for cardiovascular risk factors

a) men					
Score	number of events	number of patients	event rate (%)	hazard ratio (95% CI)	p value
0–10	2	63	3.1	1	
11–100	8	80	10.0	1.9 (1.5–2.5) *	0.04
101 – 400	23	86	26.7	5.4 (4.5–7.0) *	0.02
> 400	66	201	32.8	9.5 (8.0–11.9) *	0.01
All	99	430	10.8		

b) women					
Score	number of events	number of patients	event rate (%)	hazard ratio (95% CI)	p value

0–10	1	74	2.8	1	
11–100	5	82	6.1	1.6 (1.1–2.3) *	0.02
101 – 400	22	60	36.7	5.2 (4.0–6.9) *	0.02
> 400	35	70	50.0	14.1 (12.0–16.8) *	0.01
All	62	286	9.9		

* indicates a significant difference (p < 0.05) compared to the patient group without coronary calcifications.

**Figure 3:**
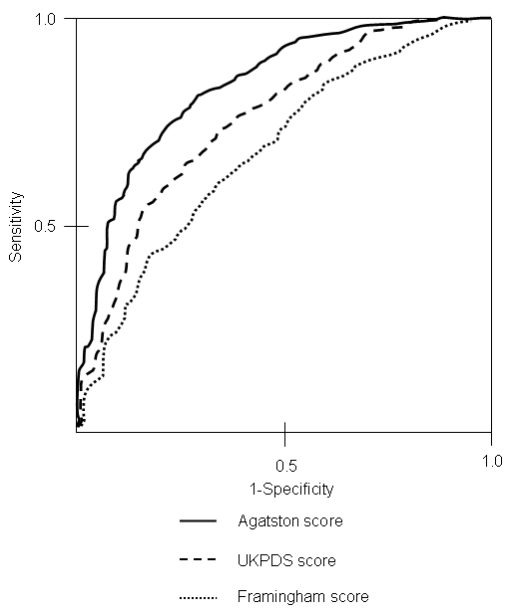
ROC curves and area under the curve of UKPDS score, Framingham score, and Agatston score for the prediction of myocardial infarction.

To illustrate the diagnostic accuracy of UKPDS score, Framingham score and Agatston score for the prediction of MI, ROC curves were calculated and the area under the ROC curve representing the diagnostic threshold was determined. The area under the ROC curve of the Agatston score was significantly larger than the area under the ROC curves of UKPDS score and Framingham score (0.76, confidence interval 0.73 – 0.82, compared to 0.63, confidence interval 0.59 – 0.66, and 0.66, confidence interval 0.62 – 0.68, p = 0.03, figure 3)

## Discussion

The aim of this study was to evaluate the predictive value of coronary calcifications in a high risk population, patients suffering from diabetes, over a long-term observation period of 8 years. The results were compared to conventional risk stratification using Framingham score and UKPDS score. On study entry all patients were not only asymptomatic, but also showed negative results in ECG, stress ECG, and echocardiography. Thus, further diagnostics or interventions were not indicated following the present guidelines and the study population could be considered a homogenous collective without evidence of coronary artery disease on study entry.

The distribution of risk factors and diabetes associated diseases is similar to study populations in prior studies examining cardiovascular risk stratification in patients with diabetes mellitus [16,21]. Compared to prior studies evaluating the predictive value of coronary calcifications the number of risk factors per person is increased, which can be explained by the preselection due to concentrating on patients with diabetes referred for a cardiological examination [12,13,22].

### Distribution of coronary calcifications

It has been discussed, if cardiovascular risk stratification can be improved by determination of coronary calcium in populations with an already increased risk for cardiovascular events such as patients with diabetes mellitus or elderly patients. The average score of 288 ± 153 in this study was higher compared to non-diabetic populations described by Hoff et al., Rumberger et al., and Schmermund et al. [23,24,22]. Still we could find a similar score distribution within our population: As described in several previous publications the mean Agatston score increased with patients' age and was significantly higher in male than in female patients. As expected, an increase of coronary calcifications in patients with additional cardiovascular risk factors could be observed. Within these subgroups a further discrimination of patients by calcium score was possible: In each group patients with a score of zero could be found, whereas in patients without additional risk factors scores up to 430 could be detected. These findings comply not only with studies in non-diabetic populations [25,12], but a similar score distribution could be found in other studies examining patients with an elevated risk of cardiovascular event and elevated calcium scores: Vliegenthart et al. achieved equivalent results in elderly patients, Anand et al. in diabetic patients [26,21].

Therefore, an additive value of coronary calcium for an improved risk stratification can be assumed in diabetic individuals.

### Cardiovascular events

This assumption is being supported by the significantly higher event rate for CR, MI, and CD of patients with an Agatston score above 400. In addition future MI or CD could be excluded in patients without coronary calcifications (table 2). This could be observed independently of concomitant risk factors in all age groups indicating that coronary calcifications reflect the patient's individual extent of atherosclerosis. Therefore, coronary calcifications can be considered as an adequate tool for the individual prediction of cardiovascular events and might be superior to conventional risk stratification.

The high accuracy of coronary calcifications is expressed by the score distribution in patients that suffered from MI during the observation period. Although we found significantly higher Framingham scores and UKPDS scores in patients with MI the risk stratification using the Agatston score was superior to Framingham score and UKPDS score: Whereas only 34% and 36% of patients with MI were classified as high risk patients according to Framingham score and UKPDS score, 85% of patients with MI were considered as high risk patients according to Agatston score. Most patients with MI, 57% and 60%, were classified as patients with an intermediate risk by Framingham score and UKPDS score. The score distribution in patients with MI was thereby comparable to prior studies in non-diabetic populations examined by Achenbach et al. and Becker et al. [12,25].

The strong correlation between coronary calcium and future cardiovascular events is also expressed by the increasing relative risk in different score groups. The relative risk in dependence of elevated Agatston score levels was significantly higher than the relative risk of conventional cardiovascular risk factors, diabetes associated diseases, or Framingham and UKPDS score (see table 3 and 4).

The high diagnostic accuracy of the Agatston score is also shown by the high negative predictive value: In all 107 patients with exclusion of coronary calcifications future cardiovascular events could be excluded. Thus, the present study with a long-term observation period in diabetic individuals could confirm the results in prior studies by Greenland et al, Achenbach et al., and Anand et al. [27,12,21].

In addition, the significantly higher discriminatory power of the Agatston score for the prediction of MI is shown by the significantly larger area under the ROC curve of the Agatston score compared to Framingham and UKPDS score (0.76 versus 0.63 and. 0.66, respectively, p = 0.03).

### Limitations

The study population consisted of patients with diabetes sent to our institution for a preventive medical check up. Therefore, the collective can not be considered as an unselected population. Still it can be regarded as a homogenous population without signs of CAD on study entry, as ECG, stress ECG, and echocardiography showed regular findings. All patients received an equivalent treatment according to current guidelines. And it might be just these patients with diabetes mellitus sent for further diagnostics that profit most from improved risk stratifications.

Even if the exclusion of future cardiovascular events by exclusion of coronary calcifications seems to be possible, it is still questionable, whether a reduction of the current prophylactic therapy in these patients is advisable. Rather a reduction of further examinations e.g. myocardial scintigraphy or coronary angiography could result from an exclusion of coronary calcifications.

Still it has to be determined, whether the detection of coronary calcification can help to establish more efficient therapies and diagnostics in patients with diabetes.

This study was performed using the EBCT, which is hardly available today. It has already been shown, that an equivalent quantification of coronary calcifications is possible by multislice computed tomography [28,29].

## Conclusion

Within a diabetic population, patients with a high risk for future MI and CD could be identified by the determination of coronary calcifications independent of concomitant cardiovascular risk factors. Thereby the Agatston score showed a higher diagnostic accuracy in predicting MI compared to Framingham risk score or UKPDS risk score. The exclusion of coronary calcifications allowed the exclusion of future cardiovascular events within a long term observation period of 8 years.

## Competing interests

The authors declare that they have no competing interests.

## Authors' contributions

AB is responsible for coordination of the study, statistical evaluation and manuscript. AL is responsible for the evaluation of coronary calcifications. CB performed EBCT scanning. FZ, JT, and IS performed examinations on study entry and follow up interviews. GS is responsible for manuscript review. AK is responsible for coordination of the study.

## Pre-publication history

The pre-publication history for this paper can be accessed here:



## References

[B1] King H, Aubert RE, Herman WH (1998). Global burden of diabetes 1995–2025: prevalence, numerical estimates and projections. Diabetes Care.

[B2] Nathan DM, Meigs J, Singer DE (1997). The epidemiology of cardiovascular disease in type 2 diabetes mellitus: how sweet it is ... or is it?. Lancet.

[B3] Juutilainen A, Lehto S, Rönnemaa T, Pyörälä K, Laakso M (2005). Type 2 diabetes as a'coronary heart disease equivalent': an 18-year prospective population-based study in Finnish subjects. Diabetes Care.

[B4] Meigs JB (2003). Epidemiology of cardiovascular disease in type II diabetes mellitus. Acta Diabetol.

[B5] Anderson KM, Castelli WP, Levy D (1987). Cholestrol and mortality: 30 years of follow-up from the Framingham study. JAMA.

[B6] Guzder RN, Gatling W, Mullee MA, Mehta RL, Byrne CD (2005). Prognostic value of the Framingham cardiovascular risk equation and the UKPDS risk engine for coronary heart disease in newly diagnosed Type 2 diabetes: results from a United Kingdom study. Diabet Med.

[B7] Akosah KO, Schaper A, Cogbill C, Schoenfeld P (2003). Preventing myocardial infarction in the young adult in the first place: how do the National Cholsterol Education Panel III guidelines perform?. J Am Coll Cardiol.

[B8] Rumberger JA, Simons DB, Fitzpatrick LA, Sheedy PF, Schwartz RS (1995). Coronary artery cacium area by electronbeam computed tomography and coronary atherosclerotic plaque area: a histopathologic correlative study. Circulation.

[B9] Mautner GC, Mautner SL, Froehlich J, Feuerstein IM, Proschan MA, Roberts WC, Doppman JL (1994). Coronary artery calcification: assessment with electron beam computed tomography and histomorphometric correlation. Radiology.

[B10] Rumberger JA, Sheedy PF, Breen JF, Schwartz RS (1997). Electron beam computed tomographic coronary calcium score cutpoints and severity of associated angiographic lumen stenosis. J Am Coll Cardiol.

[B11] Haberl R, Becker A, Leber A, Knez A, Becker C, Lang C, Brüning R, Reiser M, Steinbeck G (2001). Correlation of Coronary Calcification and Angiographically Documented Stenoses in Patients With Suspected Coronary Artery Disaese: Results of 1764 Patients. J Am Coll Cardiol.

[B12] Achenbach S, Nomayo A, Couturier G, Ropers D, Pohle K, Schlundt C, Schmermund A, Matarazzo TJ, Hoffmann U, Daniel WG, Killip T (2003). Relation Between Coronary Calcium and 10-Year Risk Scores in Primary Prevention Patients. Am J Cardiol.

[B13] Kondos G, Hoff J, Sevrukov A, Daviglus ML, Garside DB, Devries SS, Chomka EV, Liu K (2003). Electron-Beam Computed Tomography Coronary Calcium and Cardiac Events A 37-month Follow-Up of 5635 Initially Asymptomatic Low- to Intermediate-Risk Adults. Circulation.

[B14] Detrano RC, Wong ND, Doherty TM, Shavelle RM, Tang W, Gintzon LE, Budoff MJ, Narahara KA (1999). Coronary Calcium Does Not Accurately Predict Near-Term Future Coronary Events in High-Risk Adults. Circulation.

[B15] Stratton IM, Alder AI, Neil HA, Matthewes DR, Manley SE, Cull CA, Hadden D, Turner RC, Holman RR (2000). Association of glycaemia with macrovascular and microvascular complications of type II diabetes (UKPDS 35): a prospective observational study. BMJ.

[B16] World Health Organization (1999). Definition, diagnosis and classification of diabetes mellitus and its complications. Part I. Diagnosis and classification of diabetes mellitus.

[B17] Wilson PW, D'Agostino RB, Levy D, Belanger AM, Silbershatz H, Kannel WB (1999). Prediction of coronary heart disease using risk factor catagories. Circulation.

[B18] Agatston AS, Janowitz WR, Hildner FJ, Zusmer NR, Viamonte M, Detrano R (1990). Quantification of coronary artery calcium using ultrafast computed tomography. J Am Coll Cardiol.

[B19] Marwick TH, Shaw LJ, Lauer MS, Kesler K, Hachamovitch R, Heller GV, TRavin MI, Borges-Neto S, Berman DS, Miller DD (1999). The nonivasive prediction of cardiac mortality in men and women with known or suspected coronary artery disease. Economics of Noninvasive Diagnosis (END) Study Group. Am J Med.

[B20] Hanley JA, McNeil BJ (1983). A method of comparing the areas under receiver operating characteristic curves dereives from the same cases. Radiology.

[B21] Anand DV, Lim E, Hopkins D, Corder R, Shaw LJ, Sharp P, Lipkin D, Lahiri A (2006). Risk stratification in uncomplicated type 2 diabetes: prospective evaluation of the combined use of coronary artery calcium imaging and selective myocardial perfusion scintigraphy. Eur Heart J.

[B22] Schmermund A, Erbel R, Silber S, for the MUNICH Registry Study Group (2002). Age and Gender Distribution of Coronary Artery Calcium Measured by Four-Slice Computed Tomography in 2.030 Persons with no Symptoms of Coronary Artery Disease. Am J Cardiol.

[B23] Hoff J, Daviglus M, Chomka EV, Krainik AJ, Sevkurov A, Kondos GT (2003). Conventional Coronary Artery Disease Risk Factors and Coronary Artery Calcium Detected by Electron Beam Tomography in 30.908 Healthy Individuals. Ann Epidemiol.

[B24] Rumberger J, Kaufmann L (2003). A Rosetta Stone for Coronary Calcium Risk Stratification: Agatston, Volume, and Mass Scores in 11.490 Indiviuals. AJR.

[B25] Becker A, Leber A, Becker C, Knez A (2008). Predective value of coronary calcifications for future cardiac events in asymptomatic individuals. Am Heart J.

[B26] Vliegenthart R, Oudkerk M, Hofman A, Oei HH, van Dijck W, van Rooij FJ, Witteman JC (2005). Coronary calcification improves cardiovascular risk prediction in the elderly. Circulation.

[B27] Greenland P, Labree L, Azen SP, Doherty TM, Detrano RC (2004). Coronary Artery Calcium Score Combined With Framingham Score for Risk Prediction in Asymptomativ Individuals. JAMA.

[B28] Knez A, Becker C, Becker A, Leber A, White C, Reiser M, Steinbeck G (2002). Determination of coronary calcium with multi-slice spiral computed tomography: a comparative study with electron beam CT. Int J Cardiovasc Imaging.

[B29] Becker CR, Kleffel T, Crispin A, Knez A, Young J, Schoepf UJ, Haberl R, Reiser MF (2001). Coronary artery calcium measurement: agreement of multirow detector and electron beam CT. Am J Roentgenol.

